# Vanadate Influence on Metabolism of Sugar Phosphates in Fungus *Phycomyces blakesleeanus*

**DOI:** 10.1371/journal.pone.0102849

**Published:** 2014-07-18

**Authors:** Milan Žižić, Miroslav Živić, Vuk Maksimović, Marina Stanić, Strahinja Križak, Tijana Cvetić Antić, Joanna Zakrzewska

**Affiliations:** 1 Institute for Multidisciplinary Research, Department of Life Sciences, University of Belgrade, Belgrade, Serbia; 2 University of Belgrade-Faculty of Biology, Department of Physiology and Biophysics, Belgrade, Serbia; 3 Institute of General and Physical Chemistry, NMR Laboratory, Belgrade, Serbia; University of Nebraska-Lincoln, United States of America

## Abstract

The biological and chemical basis of vanadium action in fungi is relatively poorly understood. In the present study, we investigate the influence of vanadate (V^5+^) on phosphate metabolism of *Phycomyces blakesleeanus*. Addition of V^5+^ caused increase of sugar phosphates signal intensities in ^31^P NMR spectra *in* *vivo*. HPLC analysis of mycelial phosphate extracts demonstrated increased concentrations of glucose 6 phosphate, fructose 6 phosphate, fructose 1, 6 phosphate and glucose 1 phosphate after V^5+^ treatment. Influence of V^5+^ on the levels of fructose 2, 6 phosphate, glucosamine 6 phosphate and glucose 1, 6 phosphate (HPLC), and polyphosphates, UDPG and ATP (^31^P NMR) was also established. Increase of sugar phosphates content was not observed after addition of vanadyl (V^4+^), indicating that only vanadate influences its metabolism. Obtained results from *in* *vivo* experiments indicate catalytic/inhibitory vanadate action on enzymes involved in reactions of glycolysis and glycogenesis i.e., phosphoglucomutase, phosphofructokinase and glycogen phosphorylase in filamentous fungi.

## Introduction

Vanadium is widely spread, naturally occurring, multivalent transition metal, existing in nature mostly in 5+ and 4+ oxidation states. It is essential in micromolar concentrations for many eukaryotic organisms, but at higher concentrations it is toxic and can represent an environmental threat [1]. Even though both oxidation states of vanadium can alter enzyme activities, particularly those involved in splitting a phosphate ester linkage [2], such effect is attributed mainly to vanadate (V^5+^) and arises from its structural and electronic similarity to phosphate [3], [4]. This analogy is most evident in its tetrahedral, but is also valid for other V^5+^ forms [5]. Vanadate is known as a potent inhibitor of many enzymes involved in sugar phosphate metabolism such as: glucose 6 phosphatase [6], phosphoglucomutase, phosphoglyceratemutase [7], [8], fructose 2,6 bisphosphatase [7]–[9]. On the other hand, it has stimulatory effect on glucose 6 phosphate dehydrogenase, ribulose 5 phosphate epimerase, phosphoglucose isomerase and adenylyl cyclase [10].

It has been demonstrated that fungi have the ability to take up and accumulate vanadium [11]. In fact, there are a few edible vanadium-rich mushrooms useful in treatment of some types of cancer, hypertension, hypercholesterolemia and hepatitis [12]. As peculiarity, high content of vanadium was confirmed in three species of *Amanita* mushrooms [13]. In yeasts, vanadate dimmer acts as inhibitor of glucose 6 phosphate dehydrogenase the first enzyme in phosphate-pentose pathway [14], while the tetramer has inhibitory effect on 6-phosphogluconate dehydrogenase [15]. Concerning other aspects of V^5+^ influence on fungal phosphate metabolism, the role of polyphosphates (PolyP) should not be neglected. It was demonstrated that PolyP play a central role in vanadate detoxification in *Hansenula polymorpha* [16]. Also, V^5+^ addition considerably increases PolyP level in vanadate resistant yeasts by inhibiting the enzyme exopolyphosphatase [17]. Nevertheless, the data about vanadate influence on (phosphate) metabolism of fungi are very scarce and were obtained almost solely on two ascomycetous yeasts species: *Sacharomyces cerevisiae* and *H. polymorpha* [18], and especially little is known about effects of vanadate on phosphate metabolism of filamentous fungi. It should be noted that due to diversity of fungal species, the results obtained on one fungus (especially yeast), cannot be simply extrapolated to other fungal species.

The action of vanadium on metabolism of zygomycetous fungus *P. blakesleeanus,* has not been examined so far. It is known, however, that the metabolism of sugar phosphates (SP) in *P. blakesleeanus* occurs mainly through glycolysis [19], [20] and is controlled at the level of phosphofructokinase (PFK) [21]. It was demonstrated that the cessation of spores dormancy induces strong transient burst of fructose 2,6 bisphosphate (F2,6P) and hexose 6 phosphates [22]. In addition, almost all components of carbohydrate metabolism in the mycelium of *P. blakesleeanus* appear to be influenced by light growth conditions [23]. Regarding other phosphate compounds, the importance of PolyP in energy storage of *P. blakesleeanus* should be noted [24]. It was shown that its content in this fungus is dependent on stage and cultivation conditions [25].

The mechanisms of vanadium reduction, entrance into *P. blakesleeanus* in both (4+ and 5+) oxidation states, and toxicity were proposed in our previous paper [26]. We suggested that V^5+^ is physiologically active form, provoking the changes in content of phosphate compounds in mycelium. In this paper, ^31^P NMR spectroscopy and HPLC analysis were used for the investigation of vanadium influence on phosphate metabolism of *P. blakesleeanus*.

## Materials and Methods

### Mycelium cultivation and materials

The wild-type strain of the fungus *P. blakesleeanus* (Burgeff) (NRRL 1555(−)) was used. The mycelium was grown in standard minimal medium, with spore concentration of 10^6^/ml [27], in Erlenmeyer flasks which were shaken and aerated in the growth cabinet with continuous overhead white fluorescent light of 10 W/m^2^, at temperature of 20°C, and ca. 95% relative humidity. Stock solution of 200 mM sodium orthovanadate (Na_3_VO_4_) was prepared by the method of Gordon [28]. All chemicals were of analytical grade and obtained from Sigma-Aldrich (Taufkirchen, Germany).

#### ^31^P NMR

For the purpose of NMR measurements, 24 h old mycelium was collected by vacuum filtration, and washed with modified minimal medium without phosphates and microelements (experimental medium). The amount of 0.6 g of fresh weight (FW) mycelia was suspended in 2 ml of aerated experimental medium, and packed in a 10 mm diameter NMR tubes. Sodium orthovanadate (V^5+^) was added at the final concentration of 80 µmol/g_FW_. For the concentration-dependent investigation final amounts of added V^+5^ were 20, 40, 70 and 80 µmol/g_FW_; glucose 1 phosphate (G1P), glucose 6 phosphate (G6P), and fructose 6 phosphate (F6P) were added at final concentrations of 24 µmol/g, and 8-Br-cAMP at 80 µmol/g_FW_. The measurements were performed using Apollo upgrade, Bruker MSL 400 spectrometer operating at 161.978 MHz for ^31^P. Spectra were accumulated with 14 µs pulse duration (about 45°) and 300 ms recycle time. The assignment of NMR spectra and spectral line intensities evaluation were performed as described previously [25]. For ^31^P NMR analysis of mycelial extracts, the extracts prepared for HPLC experiments were used (without EDTA). SP content was estimated by NTNMR (Tecmag) software, using methylene diphosphonic acid (MDP) signal as an external standard.

### HPLC experiments

For the purpose of High Pressure Liquid Chromatography (HPLC) experiments, 24 h old mycelium was treated with V^5+^ or vanadyl sulphate (V^4+^) at final concentration of 80 µmol/g_FW_ for 10 minutes and then washed with deionised water. Control and treated mycelia were suspended in 0.5 M perchloric acid (1∶5 w/v), and homogenized in mortar on ice for 15 min. Obtained homogenate was stirred for 15 min on ice and centrifuged at 10000×g for 12 min. The precipitate was discarded and supernatant was treated with 2M KOH until pH was 7. The aliquots were kept on −20°C and unfrozen just before experiments.

HPLC analyses were performed on a Waters Breeze chromatographic system (Waters, Milford, MA) connected to Waters 2465 electrochemical detector with 3 mm gold working electrode and hydrogen referent electrode. Separation of sugars was performed on CarboPac PA1 (Dionex, Sunnyvale, CA) 250×4 mm column equipped with corresponding CarboPac PA1 guard column at constant temperature of 30°C and at a flow rate of 1.0 ml•min^−1^. Sugars were eluted using 100 mM sodium hydroxide (phase A) and 500 mM sodium acetate in 100 mM sodium hydroxide (phase B) using following gradient method: linear rise in the first 20 minutes from initial 20 to 40% B; during next 20 minutes fast rise up to 95% of phase B, followed by 15 minute reverse to 20% B with additional 5 minutes for column equilibration. Signals were detected in pulsed amperometric mode (PAD) within 150 ms of integration time using following waveform: E1 = +0.1 V for 400 ms; E2 = +0.75 V for 200 ms; E3 = −0.8 V for 180 ms. Filter timescale was 0.2 s and range was set to 500 nA for the full mV scale.

For the preparation of 100 mM NaOH solution, 5.25 ml of sodium hydroxide solution (50% w/w, low carbonate, J.T. Baker, Deventer, Holland) was diluted to final volume of 1 l with vacuum degassed deionized water. All solutions were prepared in previously vacuum-degassed deionized water. Each component was analyzed quantitatively by the external standard method using pure standards (Sigma Co. St. Louis, MO) as references for concentration and retention time. Data acquisition and quantification were carried out by Empower 2 software (Waters, Milford, MA, USA).

### Statistics

The samples were compared statistically using t-test at the 5% level of significance (P<0.05), which were performed with the Sigma Plot program, version 12.3. Data are presented as means ± s.e.m. with n indicating the number of independent experiments.

## Results

**Figure 1:**
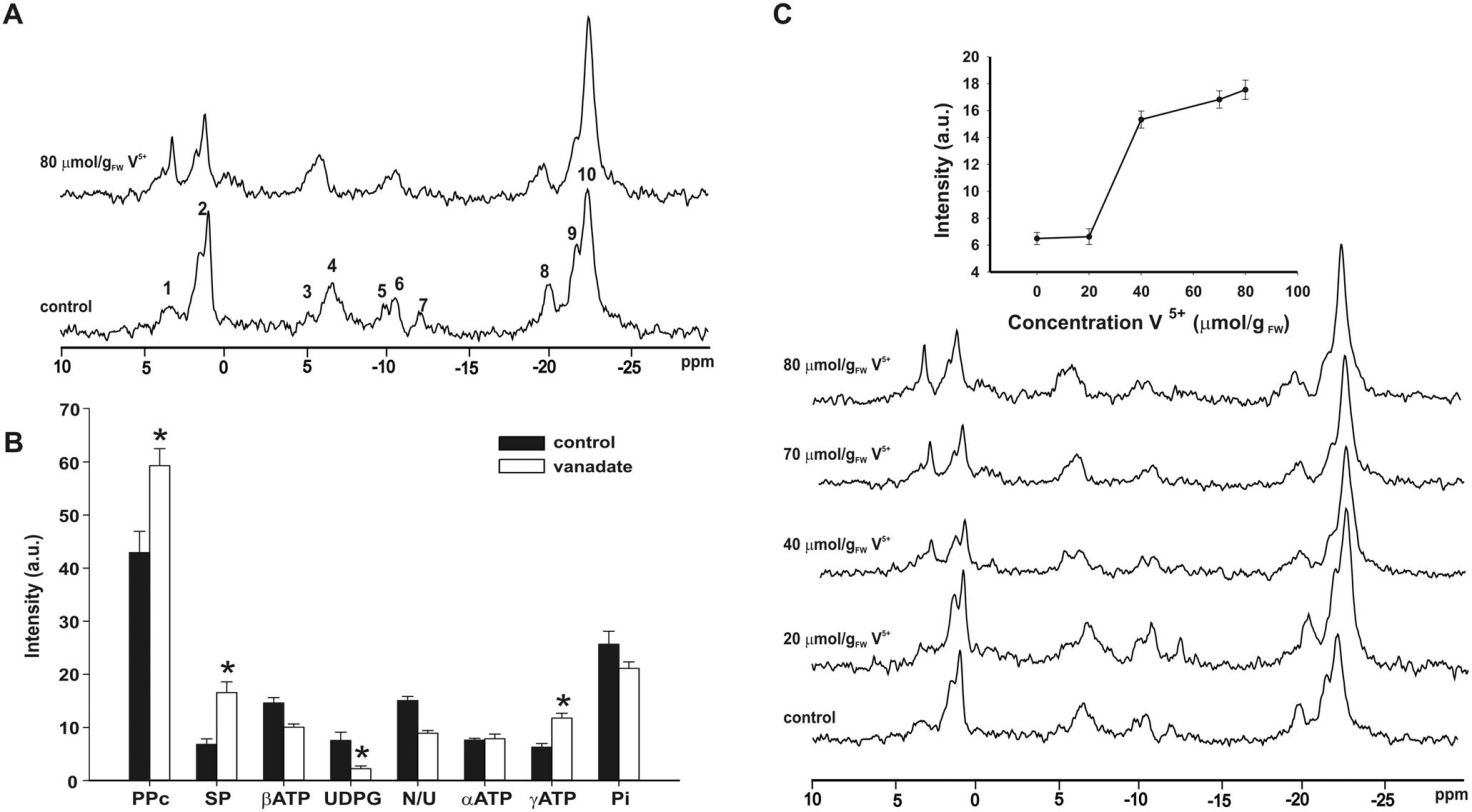
Influence of vanadate on ^31^P NMR spectrum of *P. blakeskeeanus* mycelium. **A.** Spectra of 24(control), and after addition of 80 µmol/g_FW_ V^5+^. The signals were assigned to the following compounds [25]: 1, sugar phosphate (SP); 2, inorganic phosphates (Pi); 3, γ-ATP; 4, terminal residues of PolyP and pyrophosphate; 5, α-ATP; 6, NADP(H) and UDPG; 7, UDPG (second resonance); 8, β-ATP; 9, penultimate phosphates in PolyP; 10, core PolyP residues (PPc). **B.** Signal intensities of control and 80 µmol/g_FW_ V^5+^ treated mycelium. Statistically significant differences are marked with asterix (n = 7, *P*<0.05). N/U designates NADP(H) and UDPG signal. **C.** Effect of different V^+5^ concentrations on ^31^P NMR spectrum. Insert: concentration dependency of SP signal intensity.

The ^31^P NMR spectroscopy was used to monitor the action of vanadium on the level of phosphate compounds in fungus *P. blakesleeanus*. All experiments were performed on 24 h old mycelium i.e., on mycelium in exponential phase of growth, as the effect of vanadate is most pronounced in this phase of development [29]. ^31^P NMR spectra of 24 h old mycelium before, and after addition of 80 µmol/g_FW_ V^5+^ are shown in Figure 1A. Addition of V^5+^ induced considerable changes in the intensity of almost all signals in the spectrum (Figure 1A, B), with most pronounced changes in part of the spectrum assigned to the sugar phosphates (2–5 ppm). The signals from G1P, G6P, F6P, fructose 1, 6 phosphate (F1,6P), and fructose 2, 6 phosphate (F2,6P) can be expected in this region [30]–[32]. The intensity of the broad signal centered at about 3.2 ppm increased 60%, indicating changes in SP metabolism after V^5+^ addition. Furthermore, rise in the intensity of signal at about 0.3 ppm, which can correspond to second phosphate of F2,6P [30] was noticed. The intensity of signal assigned to the core polyphosphate residues (PPc) increased by 40%, while the intensity of Pi signal decreased by 21.5%, i.e., V^5+^ caused a decrease of PPc/Pi signal intensity ratio by 67.8%. Intensity decrease (41%) of the signal originating from the phosphorus atoms of UDPG and NADP(H), together with drastic drop (71%) of signal intensity of second UDPG phosphorus indicates prominent decrease of UDPG level. Vanadate addition also affected level of ATP in the mycelium. Namely, the area of γ-ATP signal, originating from both, ATP and ADP, has increased by 43±4% (n = 7) while that of β-ATP, originating from ATP only, was reduced by 21±7% (n = 7), indicating increase of ATP consumption.

Having in mind that V^5+^ uptake occurs only when the amount of V^5+^ exceeds the reduction capacity of an enzyme with ferricyanide reductase activity [26], we examined the concentration of externally added V^5+^ required to cause the intensity increase in SP part of NMR spectrum of mycelium. Over 20 µmol/g_FW_ of externally added vanadate was needed to cause initial signal intensity increase, while the increasing concentrations of V^5+^ amplified this effect (Figure 1C).

To determine the phosphorylated sugar primarily responsible for observed intensity increase in SP part of ^31^P NMR spectrum, we performed HPLC and ^31^P NMR analysis of control and vanadate treated mycelial extracts. In ^31^P NMR spectra of mycelial extracts after V^5+^ addition, new signals, that could be assigned to G6P, F6P, F1,6P, G1P, and F2,6P (Table 1), appeared. SP contents of both, control and V^5+^ treated mycelial extracts were estimated by integration of the region from −2 to +6 ppm in ^31^P NMR spectra (SP and Pi signals area), and it was observed that this area almost doubled after V^5+^ addition.

**Table 1:** Sugar phopsphates content in perchloric acid extracts of *P. blakesleeanus* mycelium.

SP	HPLC (µmol/mg)		^31^P NMR, vanadate	
	control	vanadate	ppm	signal area*
G6P	1.915±0.090	4.380±0.211	4.3	2.45
F6P	1.001±0.209	1.651±0.106	3.7	1.34^b^
F1,6P	2.249±0.278	2.965±0.039	3.7	1.34^b^
G1P	1.098±0.106	1.448±0.087	2.5	0.31
F2,6P^a^		3.8^c^	3.7; 0.1	1.34^b^; 0.26

a compound proposed according to retention time and ^31^P NMR chemical shift.

b sum of F6P, F1,6P and F2,6P.

c relative increase of peak area normalized to the value in control.

*normalized to MDP signal area.

**Figure 2:**
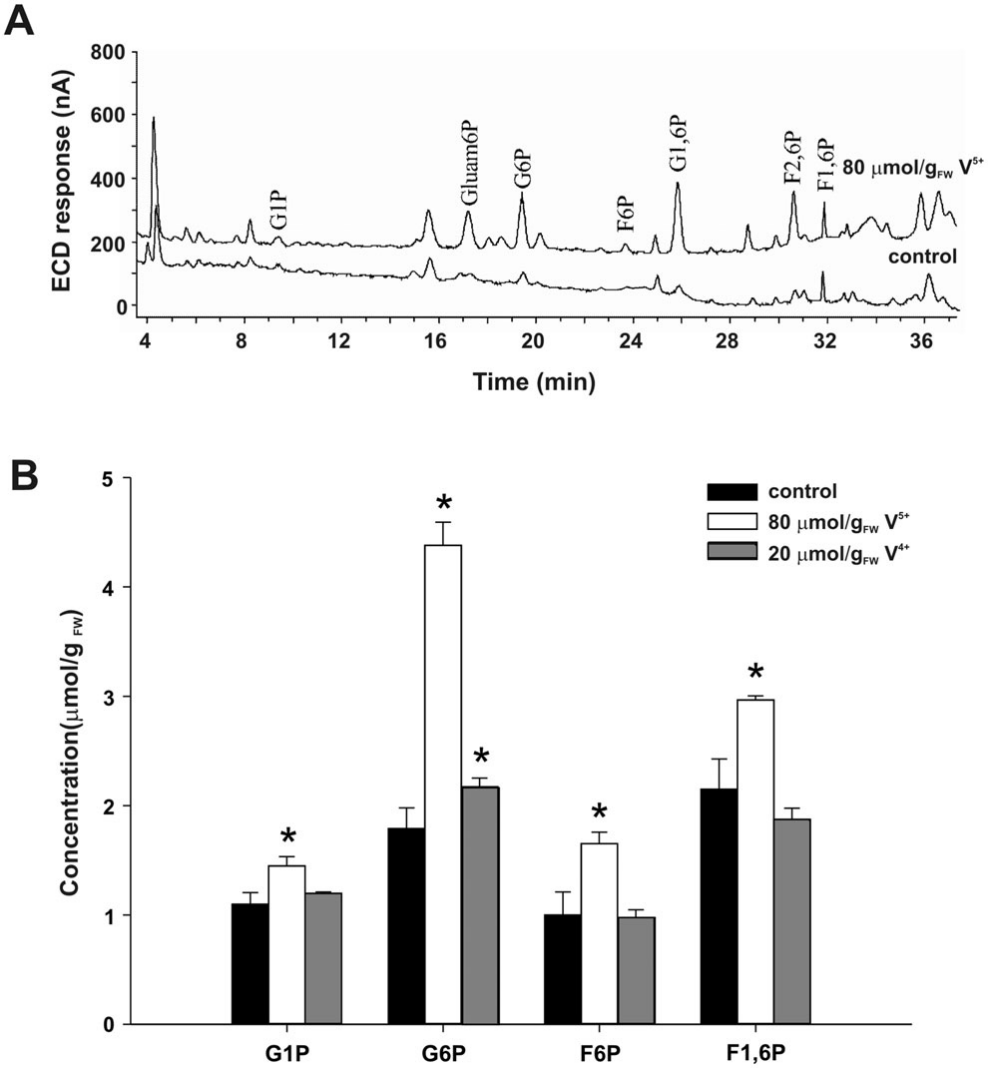
Influence of vanadium on SP content in perchloric acid extract of *P. blakeskeeanus* mycelia. **A.** HPLC chromatograms obtained from control and V^5+^ treated mycelia. **B.** Changes in content of SP caused by V^5+^, and V^4+^, and control. Statistically significant differences are marked with asterix (n = 7, *P*<0.05).

According to HPLC data (Figure 2, Table 1), V^5+^ caused the increase of G6P content by 2.3 times. Also, considerable increase was observed in contents of F6P (65%), and slightly less in F1,6P and G1P level (32%). Chromatograms of mycelial extracts after V^5+^ treatment differed from controls by noteworthy increase in the intensity of peak with retention time of 31 min, which could represent F2,6P. Furthermore, increasing intensity of peaks with retention times of 17 and 26 min that could originate from glucosamine 6 phosphate (Gluam6P) and glucose 1,6 bisphosphate (G1,6P), respectively, was observed. Lack of standards for above mentioned compounds made usual HPLC data interpretation impossible, but selectivity of HPLC-PAD method narrows down the list of interfering compounds, and makes this assignation plausible [33].

We have already proposed that the addition of vanadyl (V^4+^) has no effect on SP metabolism of *P. blakesleeanus* [26]. This statement was tested here once more, by analyzing mycelial extracts after V^4+^ treatment by HPLC (Figure 2B). In line with our previous results, almost no significant differences in chromatograms of V^4+^ treated and control mycelia were noticed.

**Figure 3:**
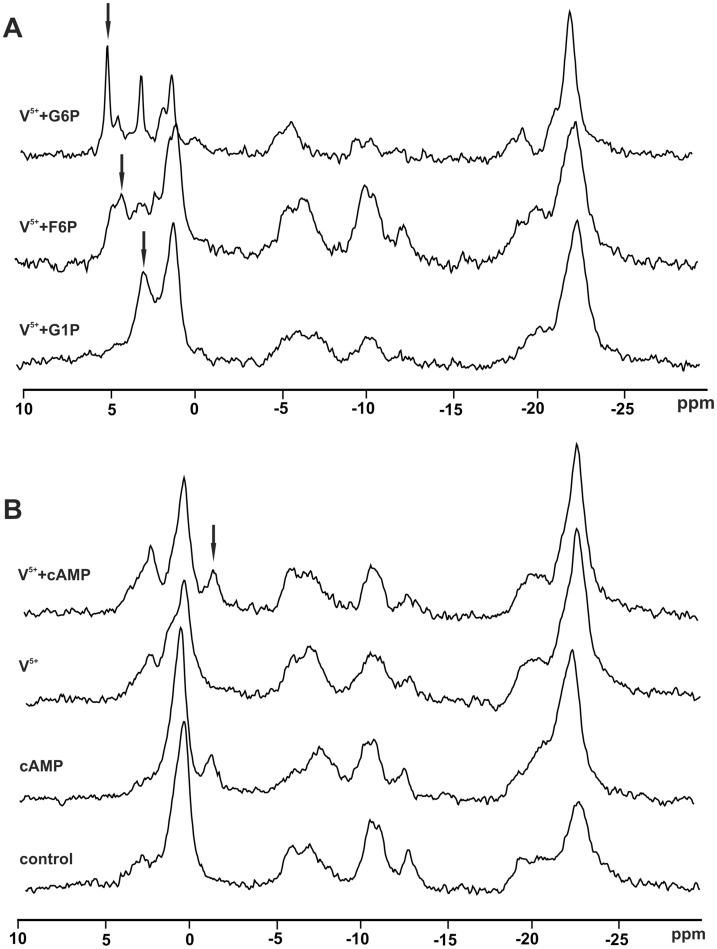
^31^P NMR spectra of *P. blakeskeeanus* mycelium after addition of: A. 24 µ**mol/g_FW_ of G6P, F6P and G1P into mycelium treated with 80** µ**mol/g_FW_ V^5+^; B. 80** µ**mol/g_FW_ cAMP, 80** µ**mol/g_FW_ V^5+^, and 80** µ**mol/g_FW_ cAMP+80** **µmol/g_FW_ V^5+^.** Positions of added compounds are labeled with arrows.

HPLC and NMR experiments performed with mycelial extracts indicate that the main effect of V^5+^ on SP content is the increase of G6P level. But, results of ^31^P NMR with fresh mycelium do not support such conclusion, since chemical shift of G6P (about 4.5 ppm) [32] is far from position of the signal most affected by V^5+^ (Figure 1C). Therefore, we examined the influence of SP addition to control and V^5+^ treated mycelium, to spike position of SP signals *in* *vivo*. After addition of G6P and F6P to V^5+^ treated mycelia, new signals appeared at 5.1 and 4.8 ppm, respectively, while the addition of G1P increased the intensity of the signal at 3.2 ppm (Figure 3A), indicating that the signal whose intensity increases mostly after V^5+^ addition belongs to G1P. Addition of the same amount of the aforementioned SP to control mycelium increased only the intensity of Pi signal. All signals originating from externally added SP disappeared after mycelium washing (results not shown) that strongly suggests that SP do not enter the cells treated with V^5+^.

Data presented so far point to the influence of V^5+^on the levels of SP involved in the metabolic reactions of glycolytic and glycogenolytic pathways. As cAMP shows considerable influence on activity of glycogen synthase, glycogen phosphorylases, and bifunctional enzyme phosphofructokinase 2/fructose 2,6 bisphosphatase in many eukaryotic organisms [34], we tested mutual action of cAMP and V^5+^. Thus, 80 µmol/g_FW_ cAMP was added before and after supplementation of mycelium with V^5+^. In both cases, addition of cAMP led only to the appearance of the signal at −0.8 ppm, that can be attributed to cAMP itself, meaning that cAMP has no effects on V^5+^ metabolic action on mycelium of *P. blakesleeanus* (Figure 3B).

## Discussion

Limited literature data on vanadium metabolism in fungi are obtained almost solely on two ascomycetous yeasts species: *S. cereviesiae* and *H. polymorpha* [18]. There is almost no data about vanadium metabolism in filamentous fungi, which make up a vast majority of fungal species, except for two studies on *Neurospora crassa* concerning vanadate uptake [35], [36], and our previous study on *P. blakesleeanus*, dealing mostly with V^5+^ reduction [26]. Influence of vanadium on fungal phosphate metabolism is even more difficult to interpret, as all available data were obtained on yeast [14]–[18]. Therefore, in this study we demonstrated the effects of V^5+^ on metabolism of phosphate compounds in filamentous fungus *P. blakesleeanus*. The action of V^5+^ was monitored by ^31^P NMR spectroscopy, method which enables detection of changes in phosphate metabolite levels *in* *vivo*, thus providing an insight to V^5+^ action. Results presented here show that V^5+^ induces changes in SP, PPc, Pi, ATP, and UDPG levels. HPLC analysis confirmed increased concentrations of G6P, F6P, G1P and F1,6P (in this order), in 24 h old mycelium of *P. blakesleeanus*.

Glucose 6 phosphate is the first product of glucose metabolism and it is further metabolized in different ways. For *P. blakesleeanus* mycelium it was shown that major part of glucose metabolism occurs via glycolysis [19]. However, considerable part of G6P in 24 h old mycelium (exponential phase) can also be involved in glycogen synthesis [37]. Namely, depending on the cultivation conditions, glycogen content can be from less than 1% to 10% of the dry mass of mycelia, reaching maximum values during the exponential growth phase [37]. The concentration of G1P after V^5+^addition increased by 36% while that of G6P by 2.3 times, according to HPLC experiments. This suggests that V^5+^ causes inhibition of the enzyme phosphoglucomutase, which participates in the first reaction of glycogen metabolism, i.e., conversion of G6P to G1P and *vice versa*, thereby preventing incorporation of G6P into glycogen. This is in accordance with the previously determined inhibitory action of vanadate on this enzyme obtained in rabbit skeletal muscle [38], [39]. Also, presumed increase in level of G1,6P is in accordance with vanadate effect on phosphoglucomutase and glucose 1,6 bisphosphate synthase in rabbit muscle [40]. The increase of Gluam6P concentration, indicated by HPLC, could be an additional proof, as the enzyme from the same group as phosphoglucomutase is responsible for the transformation of Gluam6P amino sugar to glucose-amino 1 phosphate in bacterial cells [41], and in *S. cerevisiae* [42]. All accumulated G1P could arise from glycogen degradation. Reversible process is also possible but, as can be seen from the ^31^P NMR results, the level of UDPG, main intermediate metabolite in glycogen synthesis, decreased after addition of vanadate.

Considering the stimulative effects of vanadate on cAMP accumulation, shown in rat adypocytes [43] and isolated rat liver cells [44], and consequently its influence on glycogen metabolism via glycogen phosphorylase and glycogen synthase [34], we tested the effect of cAMP on vanadate action. The accumulation of cAMP was not V^5+^-dependent, and cAMP had no effect on vanadate metabolic action in mycelium of *P. blakesleeanus*. These results are in accordance with those obtained in aquatic fungus *B. emersonii* [45]. Also, cAMP did not cause any changes in the SP region of the ^31^P NMR spectra, which were expected due to its stimulative effect on glycogen degradation determined in sporangiophores of *P. blakesleeanus* [46].

The main route of G6P transformation (glycolysis) proceeds via formation of F6P and F1,6P in reactions driven by phosphoglucoisomerase (PGI), and PFK1, respectively, the second one being irreversible. Vanadate addition caused the increase of F6P level by 65% and F1,6P by 32%. This may indicate inhibitory effect of vanadate on PFK1 activity as concentration ratio of G6P:F6P:F1,6P in control was 1∶0.5∶1.2 while, after V^5+^ treatment 1∶0.4∶0.7. However, this part of glycolysis in *P. blakesleeanus* can also include formation of F2,6P (catalyzed by PFK2), which is not a direct participant of this process [22]. Increased level of F2,6P after V^5+^addition, indicated by HPLC and ^31^P NMR data, is in agreement with formation of F2,6P established in chicken liver [7] and rat adypocytes [9], where vanadate inhibited fructose 2,6 bisphosphatase (F2,6P_2_), an enzyme which catalyses F6P synthesis through dephosphorilation of F2,6P [47]. Formation of F2,6P may also be a consequence of increased participation of glucose in glycolytic pathway caused by V^5+^ and would be in accord with previous data obtained on *P. blakesleeanus* spores [21] and *S. cerevisiae* [48], where increase in glucose concentration was directly connected with the higher content of F2,6P. It should be mentioned that glucose is present in experimental medium, giving permanent glucose input in ^31^P NMR experiments *in* *vivo*. Accumulation of F2,6P could be a trigger for the increase of F1,6P concentration. Namely, micromolar concentrations of F2,6P activate PFK1 in isolated rabbit muscle [47], *S. cerevisiae* [49] and *P. blakesleeanus* spores [21]. The noteworthy rise of F1,6P concentration, after V^5+^ addition, is in agreement with the data obtained on erythrocytes [8].

The third known way of G6P transformation is through pentose phosphate pathway, with glucose-6-P dehydrogenase (G6PD) as the first enzyme involved. Inhibition of GDPH by vanadate dimer and tetramer was demonstrated in *Leuconostoc mesenteroides* [50]. However, vanadate monomer does not show such effect in isolated baker yeast [51]. It strongly activates oxidation of glucose by G6PD, with no effects on the rates of G6P oxidation, and according to the authors, V^5+^ interacts with the 6-hydroxyl group of glucose rather than with the enzyme directly [52], [53]. The possibility of direct V^5+^ interaction with glucose, or replacement of phosphate group in G6P resulting in formation of glucose 6 vanadate (G6V), is unlikely here because significant increase of G6P level was observed after V^5+^ treatment (Table 1). Replacement of phosphate group with vanadate in NADP (cofactor in G6PD reaction) was determined in isolated baker yeast cells [51], but the level of NADP in ^31^P NMR spectra of *P. blakesleeanus* remained almost unchanged after V^5+^ treatment (Figure 1), indicating that such reaction is not plausible. Nevertheless, above mentioned modes of V^5+^ action cannot be ignored.

Some attention should be paid to the results of spiking of SP ^31^P NMR signals, which appear after V^5+^ treatment of *P. blakesleeanus* mycelium. Namely, *in* *vivo* results differ to some extent from those obtained on mycelial extracts, and suggest that the level of G1P instead of G6P is mainly influenced by V^5+^ addition. That can be explained by the fact that *in* *vivo* system is dynamic and addition of substrates/products can influence direction of glycolytic and glycogenolytic reactions, while cell extracts give insight in SP levels for a single metabolic state of the cells [54].

As it was already said, washing of mycelium after subsequent V^5+^ and SP addition, resulted in disappearance of ^31^P NMR signals attributed to added SP. Fungi are known to be absorptive heterotrophs, meaning that SP should be previously digested/hydrolyzed, and then ingested as inorganic Pi and sugar. Presence of acid phosphatase, enzyme(s) that hydrolyze phosphate group, was confirmed in yeast and mycorrhizal fungi, when inorganic phosphate was unavailable in its simplest form [55]. Based on database search, there are two protein sequences in *P. blakeesleanus* protein database which show homology with acid phosphatase protein isolated from *Penicillium chrysogenum*. Predicted gene models for these proteins are located in *P. blakeesleanus* genome at the scaffold_8∶1745265-1746626 and scaffold_2∶2647390-2648609 [56]. These sequences also show high identity (40%) with acid phosphatase from *Laccaria bicolor* S238N-H82 [57], and putative acid phosphatase from *Aspergillus fumigatus* Af293 (43%) [58]. Absence of additional signals in SP part of ^31^P NMR spectra of control and their appearance in V^5+^ treated mycelium after SP addition, strongly suggests that V^5+^ influences the activity of acid phosphatases in *P. blakesesleeanus*.

^31^P NMR results presented here also show that V^5+^ causes considerable increase of PPc signal intensity (PolyP level). Similar effects of V^5+^ were described in amoeba *Dyctyostelium discoideum* [59] and *H. polymorpha* [18]. Inhibition of exopolyphosphatase by V^5+^ was confirmed in yeast [17], [60], and could be responsible for increase of PolyP level observed here. Predicted gene model for exopolyphosphatase in *P. blakesleeanus* genome is located at scaffold_6: 2343311-2344745. Protein sequence of this enzyme shows the highest identity level (42%) with *Mucor circinelloides* hypothetical protein with activity of inorganic pyrophosphatase/exopolyphosphatase (GenBank accession number: EPB83848) [52]. Using this protein sequence as query and BLAST [61] for alignment with sequence of purified exopolyphosphatase encoded by PPX1 gene from *S. cerevisiae* [62], [63], possible homology of these two proteins was obtained (95% of query coverage, 30% of identity and E-value = 10^−40^). Since only a part of added/intracellular V^5+^ is reduced to V^4+^ [26] we propose that remaining V^5+^ induces increase of PolyP level due to inhibition of exopolyphosphatase(s) in *P. blakesleeanus*.

To the best of our knowledge, these are the first data about vanadate action on any aspect of metabolism in filamentous fungi. The action of V^5+^ on SP content and phosphate metabolism in general was studied mainly *in* *vitro* i.e., on isolated tissues of various types of organisms, or by measuring the enzyme activity. Here, based on increased intensities of SP ^31^P NMR signals *in* *vivo* and HPLC experiments, we presumed the influence of V^5+^ on enzymes involved in glycolysis and glucogenesis. Furthermore, results of ^31^P NMR indicate V^5+^ induced changes in actions of enzymes acid phosphatase and exopolyphosphatase whose presence is yet to be confirmed in *P. blakesleanus*.
